# Grafting red clay with Bi_2_O_3_ nanoparticles into epoxy resin for gamma-ray shielding applications

**DOI:** 10.1038/s41598-023-32522-7

**Published:** 2023-04-04

**Authors:** Mohamed. Elsafi, Aljawhara H. Almuqrin, Haifa M. Almutairi, Wafa M. Al-Saleh, M. I. Sayyed

**Affiliations:** 1 Physics Department, Faculty of Science, Alexandria University, Alexandria, 21511 Egypt; 2 Department of Physics, College of Science, Princess Nourah Bint Abdulrahman University, P.O. Box 84428, Riyadh, 11671 Saudi Arabia; 3 Medical Physics Department, Umm Al-Qura University, Prince Sultan Bin Abdul-Aziz Road, Mecca, Saudi Arabia; 4 College of Science and Health Professions, King Saud Bin Abdulaziz University for Health Sciences, P.O.Box 6664, Hofuf, 31982 Al-Ahsa Saudi Arabia; 5 King Abdullah International Medical Research Center, Hofuf, Al-Ahsa Saudi Arabia; 6 Department of Physics, Faculty of Science, Isra University, Amman, Jordan; 7 Department of Nuclear Medicine Research, Institute for Research and Medical Consultations (IRMC), Imam Abdulrahman Bin Faisal University (IAU), P.O. Box 1982, Dammam, 31441 Saudi Arabia

**Keywords:** Physics, Applied physics, Nuclear physics

## Abstract

We developed new composites for photons shielding applications. The composite were prepared with epoxy resin, red clay and bismuth oxide nanoparticles (Bi_2_O_3_ NPs). In order to establish which ratio of red clay to Bi_2_O_3_ NPs provides the best shielding capabilities, several different ratios of red clay to Bi_2_O_3_ NPs were tested. The transmission factor (TF) was calculated for two different thicknesses of each sample. From the TF data, we found that epoxy resin materials have a high attenuation capacity at low energy. For ERB-10 sample (40%Epoxy + 50% Red clay + 10% Bi_2_O_3_ NPs), the TF values are 52.3% and 14.3% for thicknesses of 0.5 and 1.5 cm (at 0.06 MeV). The composite which contains the maximum amount of Bi_2_O_3_ nanoparticles (40%Epoxy + 50% Red clay + 10% Bi_2_O_3_ NPs, coded as ERB-30) has lower TF than the other composites. The TF data demonstrated that ERB-30 is capable of producing more effective attenuation from gamma rays. We also determined the linear attenuation coefficient (LAC) for the prepared composites and we found that the LAC increases for a given energy in proportion to the Bi_2_O_3_ NPs ratio. For the ERB-0 (free Bi_2_O_3_ NPs), the LAC at 0.662 MeV is 0.143 cm^−1^, and it increases to 0.805 cm^−1^ when 10% of Bi_2_O_3_ NPs is added to the epoxy resin composite. The half value layer (HVL) results showed that the thickness necessary to shield that photons to its half intensity can be significantly lowered by increasing the weight fraction of the Bi_2_O_3_ NPs in the epoxy resin composite from 0 to 30%. The HVL for ERB-20 and ERB-30 were compared with other materials such as (Epoxy as a matrix material and Al_2_O_3_, Fe_2_O_3_, MgO and ZrO_2_ as filler oxides in the matrix at 0.662 MeV. The HVL values for ERB-20 and ERB-30 are 4.385 and 3.988 cm and this is lower than all the selected epoxy polymers.

## Introduction

The development of applications that make use of radiation sources has significantly increased in tandem with the general rise in the level of technical sophistication. Gamma and X-rays are examples of ionizing radiation types that are extensively used in a range of disciplines, such as the generation of nuclear power, diagnostic imaging, hospital treatment, agriculture, academic research, and other sectors. Even though radiation has many applications, especially within the medical profession, it is a well-known fact that extended exposure to radiation can have a negative impact on both the health of humans and other living things^1–4^. Due to the outstanding shielding qualities that lead and materials containing lead provide, lead and lead-containing compounds have historically been utilized extensively in the field of radiation protection. However, lead is a hazardous chemical. As a direct consequence of this, the advancement and research of practical non-toxic compounds for use in radiation shielding is currently more important than it has ever been^5–7^. Investigators are exerting a lot of effort to develop new materials with efficient radiation shielding qualities. Some examples of these new materials include ceramics, alloys, glasses, construction materials and polymer composites. By changing the composition of already-existing materials, novel shielding materials can also be made^8–11^. According to studies, adding nanoparticles to specific materials can increase their potential to shield^12,13^.

On the other hand, due to its special combination of qualities, epoxy resin is a useful and significant substance in many sectors. One of the most significant characteristics of epoxy resin is its high strength and durability. It also has superior electrical insulating capabilities, high thermal stability, and good resistance to chemicals. Due to their distinctive combination of characteristics, epoxy resins are utilized in a variety of industries. Epoxy resins are frequently used in the following industries: building, civil engineering (to repair and reinforce concrete structures), dentistry and orthopedic, as well as electrical and electronic industries. Epoxy resin can be utilized as a matrix to form composites containing nanoparticles to enhance shielding capabilities in the field of radiation protection^14–16^. Nanoparticles formed of bismuth oxide (Bi_2_O_3_) are frequently employed in radiation shielding because of their special combination of characteristics, which allows them to effectively absorb radiation. Recent studies have demonstrated that the radiation shielding ability of materials that contain Bi_2_O_3_ nanoparticles is superior to that of bulk Bi_2_O_3_ in terms of both efficiency and effectiveness^17–19^. Bi_2_O_3_ has a high melting point, which enables it to remain stable even when subjected to high temperatures and making it appropriate for usage in a wide variety of contexts. Bi_2_O_3_ is a substance that, in comparison to other compounds used for radiation shielding, such as lead, is generally non-toxic. Bi_2_O_3_ is a cost-effective alternative for radiation shielding because it is comparatively cheap in comparison to other materials that are used in the industry^20–25^.

Red clay is a naturally occurring substance that mostly consists of hydrous aluminum silicates. It is possible to increase the composite material's mechanical qualities by incorporating red clay into an epoxy matrix. Clay particles have the potential to serve as reinforcing fillers, which will result in an increase in the composite's strength and stiffness. The composite's thermal stability, flame resistance, and electrical insulating qualities can all be improved by the inclusion of clay. Investigations have been conducted into the use of red clay-epoxy composites for a variety of purposes, such as the prevention of electromagnetic interference, the management of thermal energy, and the shielding of radiation. Hence, adding red clay and nanoparticles of Bi_2_O_3_ to an epoxy matrix can enhance the composite material's physical, thermal, and electrical features, making it a potentially useful material for radiation shielding^13,26,27^. In this work, four samples based on epoxy resin as a matrix material and red clay as well as Bi_2_O_3_-NPs as a filler materials were prepared and the photons shielding performance for these materials were studied. The study was applied experimentally using high purity germanium (HPGe-detector) and different radioactive sources. The linear attenuation coefficient (LAC) was determined experimentally at 0.06, 0.662, 1.173 and 1.333 MeV for all epoxy samples. The other shielding parameters such as HVL, mean free path (MFP) and tenth value layer (TVL) were calculated.

## Materials and methods

### Materials

#### Epoxy resin (ER)

Epoxy resin (ER) with density 1.05 g/cm^3^, compressive strength 90–100 N/mm^2^, tensile strength 20–30 N/mm^2^ and flexural Strength 55–70 N/mm^2^ was used as a matrix viscous liquid material^28,29^. The ER molecular formula is C_21_H_25_ClO_5_, and the ER consists of two materials which called basic epoxy material and the hardener, the combination between them gives the ER. In the present work, the hardener liquid material is added to the composite at a percentage of 5% of the basic epoxy material added^30–32^.

#### Red clay

**Figure 1:**
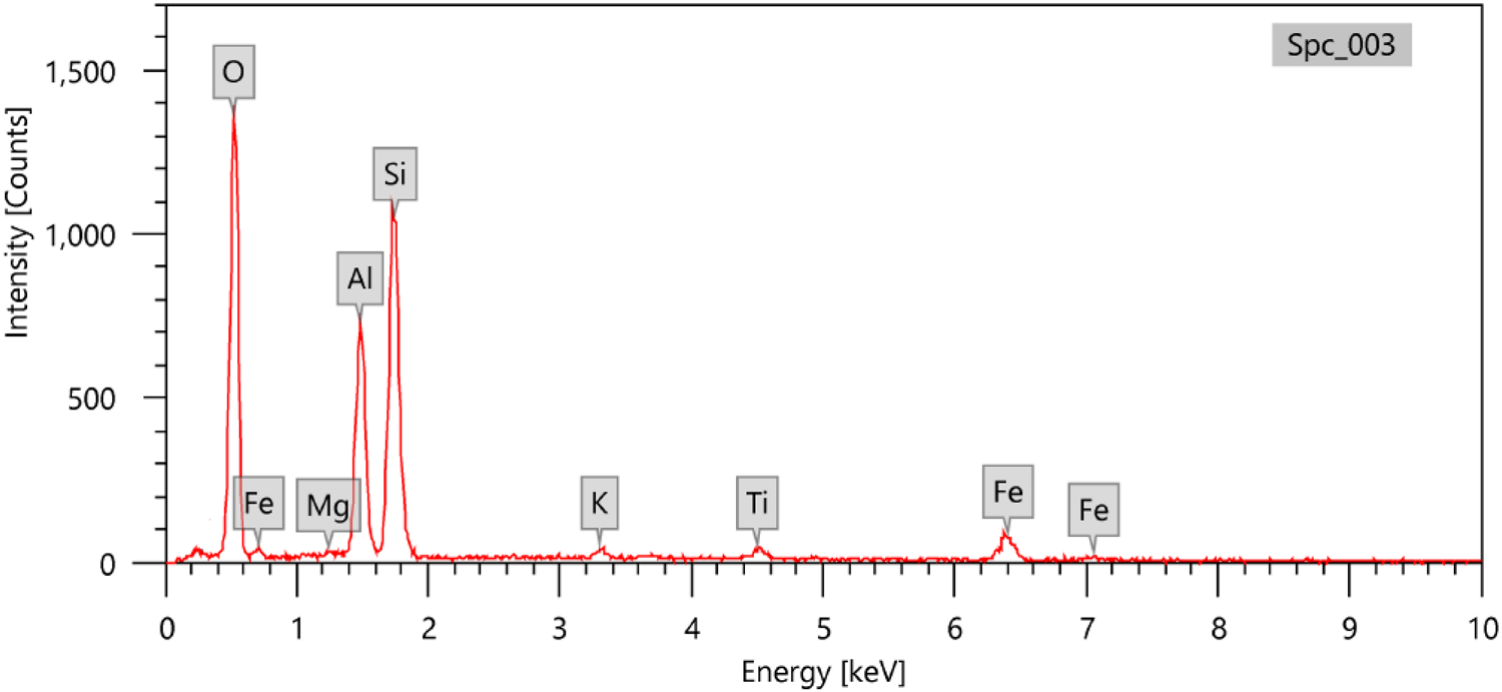
EDX analysis of red clay.

The red clay was collected from Aswan in southern Egypt. It was a fine and coarse aggregate together, so the aggregate was ground further and sifted with a 60-micron sieve, and dried under the influence of a temperature of 110 °C for two hours. Red clay was analyzed for its components and percentages using EDX analysis, as shown in Fig. 1. The figure shows the components of red clay and its high content of alumina “Al_2_O_3_” and silica in addition to some other elements, as shown in Table 1. The red clay contributed to the compound for two important reasons. The first reason is that bismuth oxide does not precipitate at the bottom of the mixture due to its high density. The second reason is that no bubbles are formed in the mixture during preparation. This is in addition to the percentage of aluminum, which in turn works on the cohesion and strength of the mixture.

**Table 1:** The chemical compositions of red clay.

Element	MgO	Al_2_O_3_	SiO_2_	K_2_O	TiO_2_	FeO	L.I.O
Percentage (%)	0.47 ± 0.10	28.05 ± 0.72	55.57 ± 0.31	1.16 ± 0.21	2.34 ± 0.18	9.08 ± 0.21	3.14 ± 0.12

#### *Bi*_*2*_*O*_*3*_* nanoparticles*

**Figure 2:**
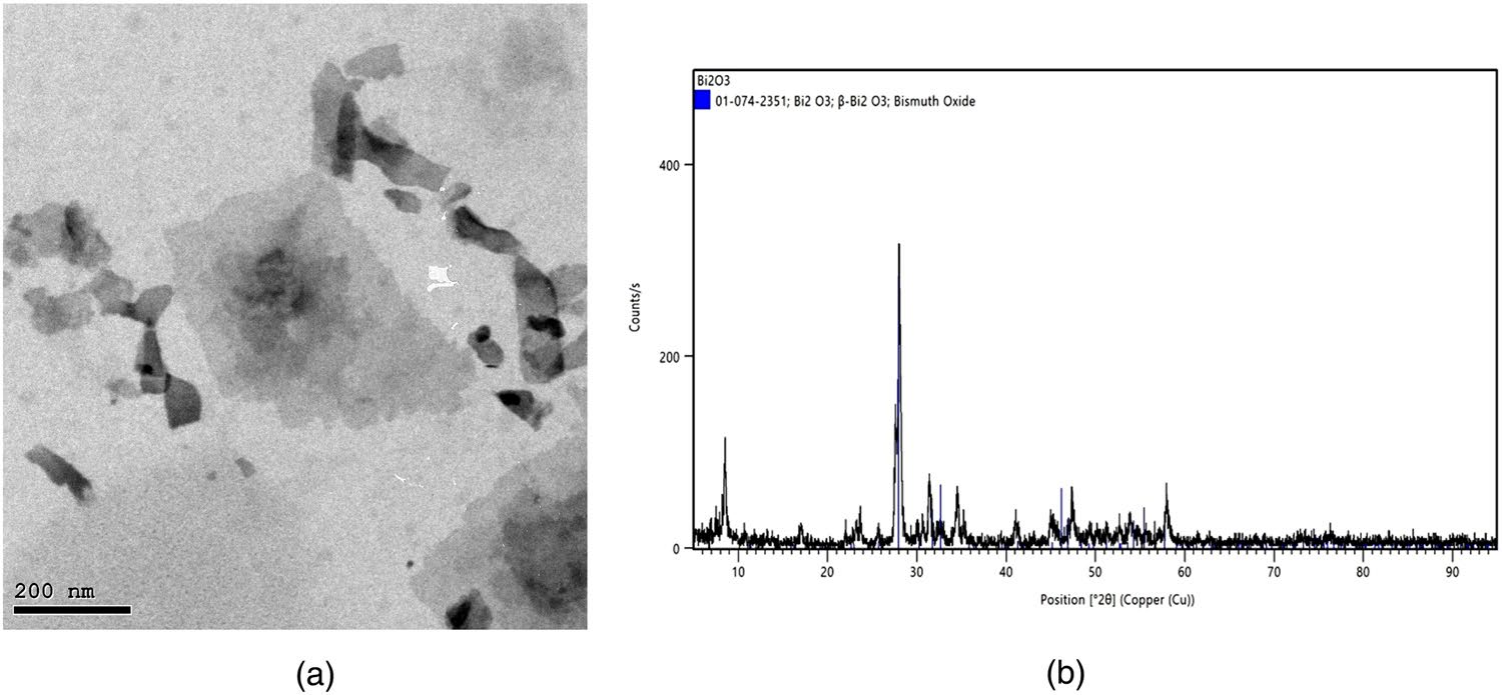
(**a**) TEM image of Bi_2_O_3_ nanoparticles and (**b**) XRD of WO_3_ nanoparticles.

Bi2O3-NPs were purchased from Nano-Gate Chemical Company, which chemically prepared them^33^. The Bi_2_O_3_ NPs powder was scanned by TEM to find out the average particle size (average 20 ± 5 nm) as shown in Fig. 2a, in addition to XRD analysis to prove the crystalline and structure of Bi_2_O_3_-NPs as shown in Fig. 2b, where the X-ray diffraction pattern of Bi_2_O_3_ materials showed reflection peaks with an angle of 31.923°. All reflection peaks can be well indexed to pure tetragonal phase of crystalline Bi2O3, which correspond well to the bismuth trioxide structure. The broad reflection peaks indicate that the material is a nanocrystalline structure and the size of Bi_2_O_3_ NPs is about 20 nm, which indicates that the product consists of spherical-shaped nanocrystals. The reason for choosing Bi2O3 NPs as filler is that in addition to the high density of Bi2O3 NPs, it has a high absorption point (k-edge) with energy of 88 keV, which affects the absorption rate in this region as well as the distribution of nanoparticles within the mixture.

### Methods

#### Mixture formulation

Four epoxy samples ERB-0, ERB-10, ERB-20, and ERB-30 were investigated according the percentages tabulated in Table.2. Each composite was weighed and putted in a suitable container, and the composite was stirred until becomes more homogeneous. The homogeneous composite after that placed in plastic molds and left to dry for two days. The prepared epoxy samples were extracted from the molds and the attenuation coefficients were calculated according to below section. The prepared samples were in disc shape with diameter 2 cm and different thickness 0.5, 1 and 1.5 cm and measured experimentally using the collimator to obtain narrow incident beam. The density was calculated by the equation $\rho =M/V$, where M is the mass of the composite and weighted in 0.001 g sensitive electronic balance and V is the volume of sample and calculated by $4/3\pi {r}^{3}$, where r is the radius of the disc sample.

**Table 2:** Chemical compositions of Epoxy resin –Red clay- Bi_2_O_3_ NPs composites.

Mixture Code	Percentage (wt%)			Density (g cm^−3^)
	Epoxy Resin	Red Clay	Bi2O3-NPs	
ERB-0	40	60	0	1.673
ERB-10	40	50	10	1.768
ERB-20	40	40	20	1.874
ERB-30	40	30	30	1.993

#### Shielding parameters

**Figure 3:**
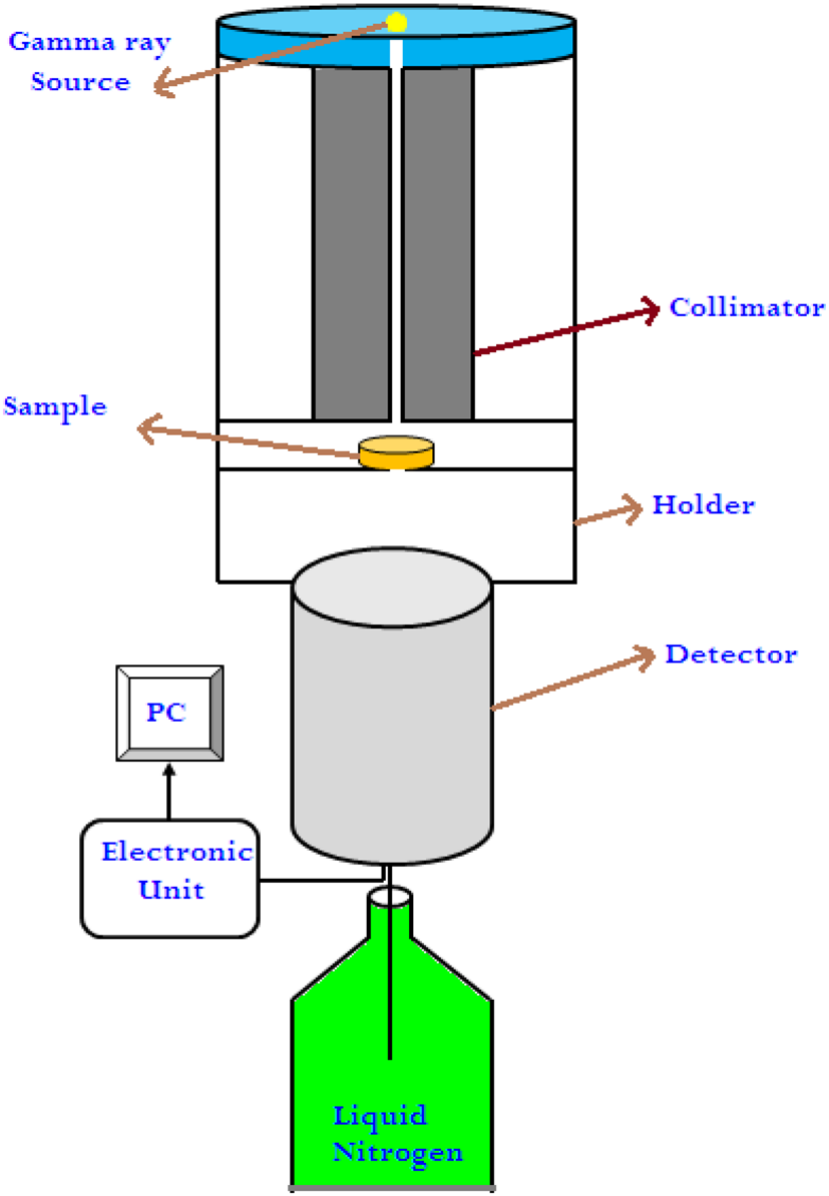
The experimental attenuation calculation geometry in the current work.

The radiation shielding parameters for the present epoxy composites were experimentally measured by HPGe detector (with Relative Efficiency 24% and 1.93 keV at 1333 keV Energy Resolution) and Co-60 (decayed with two energies 1.173 and 1.333 MeV), Am-241 (decayed with one energy 0.060 MeV) and Cs-137 (decayed with one gamma energy 0.662 MeV) three γ-ray point sources. The source was putted axially at 20 cm height from the detector top, and the collimator from lead material with 8 and 70 mm inner and outer diameter, respectively was utilized in-between the detector and the source to get a narrow beam. The source- collimator- detector setup was shown in Fig. 3. After detector calibration, the detector was run at certain time (sufficient to obtain error in peak area less than 1%) and the peak related to the incident photon energy will produce using Genei-2000 software connected to the detector. From the obtain peak, the area under this peak can be estimated (A_0_). To obtain the LAC at this energy, the sample will place between the detector and the source as shown in Fig. 3, the run happen at the same time and the area under the peak was calculated (A).

The Linear-attenuation factor can be defined as the probability of photons interaction through certain thickness of sample and experimentally estimated by knowing Area with an absorber (A) and Area without an absorber (A_0_) values using the following relation^34–36^:

1 $$LAC=\frac{1}{x}\mathrm{ln}\frac{{A}_{0}}{A}$$

where, $x$ is the layer thickness or height of the present epoxy sample. Also, the transmission factor (TF) which defines as the percentage of the intensity of gamma ray photon in the pretense and the absence of the epoxy sample ($I/{I}_{0}$) and must be evaluated by the next equation^37–39^:

2 $$TF=\frac{I}{{I}_{0}}=\frac{A}{{A}_{0}}$$

The HVL (the thickness required to absorb photon intensity by 50%), MFP (the pathelength of photon without interaction) and RSE (the radiation shielding efficiency and it measure the efficiency the epoxy materials for attenuation ability.) are important coefficients and their evaluation gives essential indications of the epoxy material's capability to absorb the gamma rays. The results of them were estimated by the following relationships^40–42^:

3 $$HVL(E)=\frac{Ln (2)}{LAC}, MFP(E)=\frac{1}{LAC}$$

4 $$RSE, \%=[1-\frac{A}{{A}_{0}}]\times 100$$

## Results and discussion

This section presents the measured attenuation characteristics and discusses the impact of altering polymers epoxy resin composites with various amounts of red clay and Bi_2_O_3_ nanoparticles (NPs). In the current work, experimental evaluations of shielding parameters including the transmission factor (TF), linear and linear attenuation coefficients have been conducted. From the results, we can examine the impact of thickness of the prepared ERB-0, -1, ERB-20 and ERB-30 composites on the TF. Also, we can understand the impact of changing the amount of red clay and Bi_2_O_3_ NPs on the LAC (and the other shielding parameters). It is important to remember that an increase in the proportion of Bi_2_O_3_ NPs on the expense of red clay in the composite leads to an increase in the density of the net composite.

**Figure 4:**
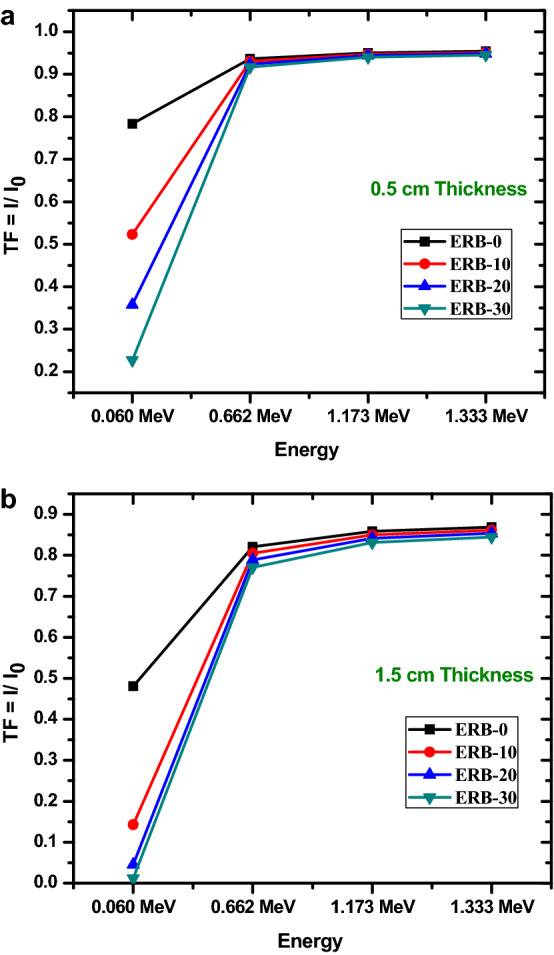
(**a**) The transmission factor for the prepared composites with thickness 0.5 cm. (**b**) The transmission factor for the prepared composites with thickness 1.5 cm.

The transmission factor, or TF, is a standard method for estimating the specific amount of photons that can pass through the attenuator. It provides a measure of the total amount of photons that the composite samples have the potential to absorb, reflect, or scatter. We were able to determine the TF for the ERB-0, ERB-10, ERB-20 and ERB-30 composites at the energies that were studied (between 0.06 and 1.333 MeV) by using the intensities of the photons that were entering and leaving the sample. We studied the relation between TF for the prepared materials with the thickness in Fig. 4a,b. In Fig. 4a, we showed the results for a thickness of 0.5 cm, where the measurements were done in the absence and the presence of 0.5 cm epoxy absorber, while in Fig. 4b, we showed the results for a thickness of 1.5 cm. The ERB-30 sample had the lowest TF at the lowest energy, which was 0.06 MeV. It had a value of 22.7% when the thickness was 0.5 cm, and it had a value of 1.2% when the thickness was 1.5 cm. From the TF data, it can be inferred that epoxy resin materials have a high attenuation capacity at low energy. From Fig. 4a, the TF for the ERB-10 sample at 0.06 MeV is 52.3%, which implies that only 52.3% of the incoming photons can pass through this sample because it can protect 47.7% of them. We can see from Fig. 4a and b that the TF reduces as the thickness increases. To put it another way, the number of gamma rays that are able to pass through the shield at any given thickness drops in a manner that is exponentially proportional to the thickness of the shield. For example, for ERB-10, the TF values are 52.3% and 14.3% for thicknesses of 0.5 and 1.5 cm (at 0.06 MeV). For the same sample and for these two selected thicknesses, the TF values at 0.662 MeV are 93 and 85.5%. This pattern can be summarized as follows: the thicker the epoxy resin composite is, the more gamma radiation are absorbed by the shields before they are able to pass through to the opposite side. The amount of atoms that the photons must pass through as the shield's thickness rises increases the likelihood that they will be absorbed, which causes the TF to fall.

In addition to this, it has been observed that the TF of the ERB-30 sample is significantly lower than the TF of the other composites. The higher percentage of Bi_2_O_3_ nanoparticles in ERB-30 composite is the cause of the lower TF that it possesses. This demonstrates that ERB-30 is capable of producing more effective attenuation from gamma rays. In addition, the TF data demonstrate that for 1.173 and 1.333 MeV, all of the fabricated nanocomposites have high TF values. This is shown for all of the composites. This demonstrated that all of these different types of materials are more effective than others at reducing the low-energy photons.

**Figure 5:**
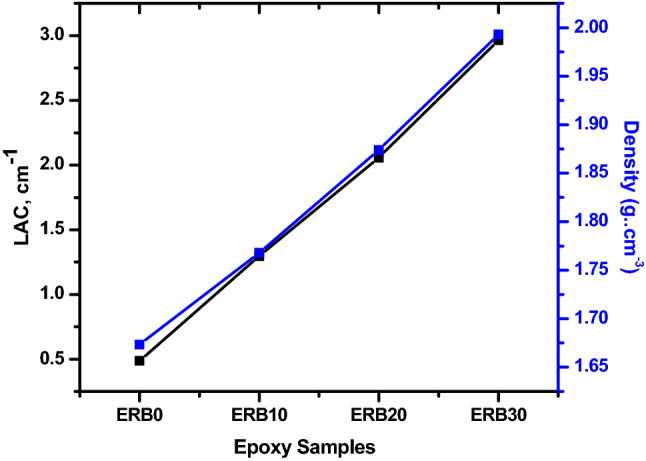
The relation between the linear attenuation coefficient and the density of the prepared composites at 0.06 MeV.

From the TF and the thickness, and with the help of Lambert–Beer law, we evaluated the linear attenuation coefficient for each composite. In Fig. 5, we exhibited the LAC at 0.060 MeV and the density for each composite. It is clear that both parameters are increasing in Fig. 5, confirming the increase in the LAC for the prepared nanocomposites with increasing the density. On the other words, when a composite is denser, it indicates that it contains a greater number of atoms packed into a given volume; as a result, there is a greater likelihood that gamma rays will be absorbed by the composite. Therefore, as the density of the composite grows, the absorption probability increases while the transmission probability drops (as we found in the previous curves); this is the reason why the LAC increases as the density of the current composite increases.

**Figure 6:**
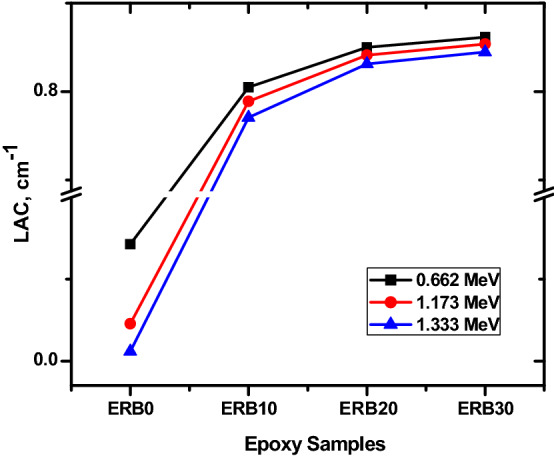
The linear attenuation coefficient for the prepared composites.

The variations of the LAC at 0.662, 1.173 and 1.333 MeV for the ERB-0, ERB-10, ERB-20 and ERB-30 composites is plotted in Fig. 6. The LAC increases for a given energy in proportion to the Bi_2_O_3_ Nps ratio. Numerically, For the ERB-0 (free Bi_2_O_3_ NPs), the LAC at 0.662 MeV is 0.143 cm^−1^, and this parameter increases to 0.805 cm^−1^ when 10% of Bi_2_O_3_ NPs is added to the epoxy resin composite. At 0.662 MeV, the maximum LAC if found for ERB-30 (equivalent to 0.862 cm^−1^). Hence, at 0.662 MeV, increase the Bi_2_O_3_ NPs content by 30% leads to 6 times enhancement in the LAC. At 1.173 MeV, the LAC increases from 0.046 cm^−1^ (for free Bi_2_O_3_ NPs) to 0.854 cm^−1^. According to these values, it was concluded that increasing the proportion of doped material (i.e. Bi_2_O_3_ NPs) by 30% led to an improvement in the gamma ray attenuation. The LAC reached their best value in the scenario in which 30% of the composite was composed of Bi_2_O_3_ NPs (i.e. for ERB-30 composite).

**Figure 7:**
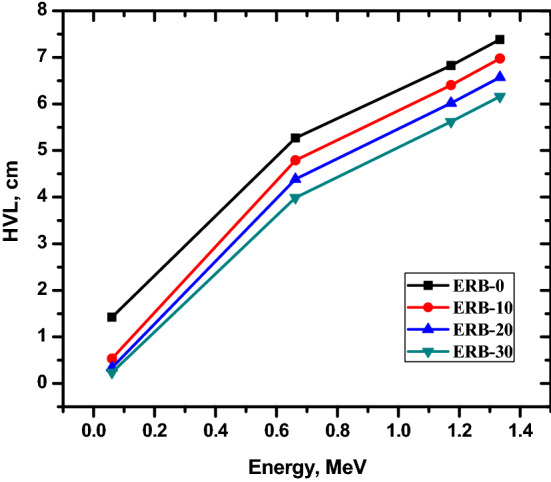
The half value layer for the prepared composites.

**Figure 8:**
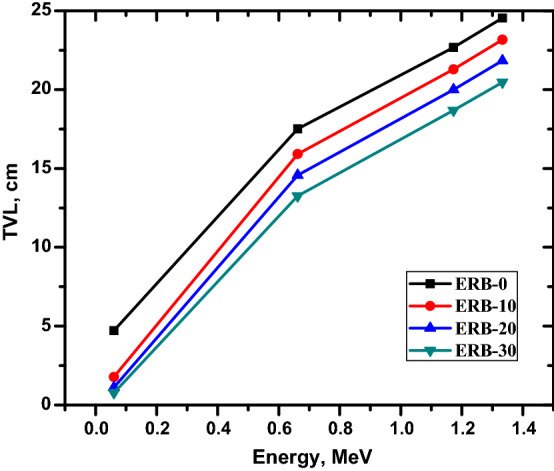
The tenth value layer for the prepared composites.

The half and tenth value layers are depicted in Figs. 7 and 8 as functions of gamma energy. The TVL and HVL of the shielding material are major indicators of the protecting effectiveness and for this reason they have been looked into in the present study. The lower the HVL, the less material is needed to attenuate the photons to half of its initial intensity. At 0.06 MeV, the lowest HVL for the ERB-0, ERB-10, ERB-20 and ERB-30 that were tested was recorded. The HVL for that particular energy varies from 0.234 cm for the ERB-30 composite to 1.420 cm for the ERB-0 composite. This finding suggests that the thickness necessary to shield that photons to its half intensity can be significantly lowered by increasing the weight fraction of the Bi_2_O_3_ NPs in the epoxy resin composite from 0 to 30%. The effectiveness of raising the Bi_2_O_3_ NPs ratio on reducing the HVL of the present composites lowers toward the other energies selected in this work. The reason for this is that as the energy of the gamma rays increases, the wavelength decreases, resulting in a greater ability to penetrate through the present composites. For example, the radiation with energy of 0.662 MeV can be attenuated to 50% of its original level by utilizing a shield thickness of 5.271 and 3.988 cm of the ERB-0 and REB-30 respectively.

The same relation between the shielding ability and the Bi_2_O_3_ NPs ratio can be seen in Fig. 8. This figure shows that increasing the Bi_2_O_3_ NPs ratio from 0 to 30% causes a reduction in the TVL from 4.717 to 0.776 cm at 0.06 MeV and from 17.51 to 13.249 cm at 0.662 MeV. Epoxy resin composites that are strengthened with Bi2O3 nanoparticles can be drawn to the conclusion that they have the potential to become a promising shielding material. This type of composite has the benefits of high-Z constituent elements, such as bismuth, but does not make use of lead. Additionally, because of the tested composite's very low mass density, extremely low toxicity, and excellent conformability, its shielding abilities have a lot of potential for usage in the healthcare sector.

**Figure 9:**
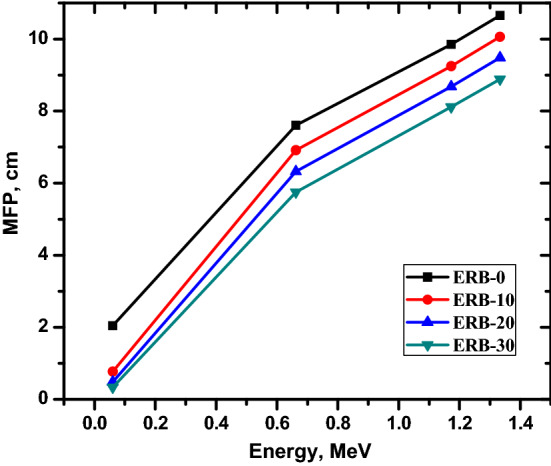
The mean free path for the prepared composites.

Determining the MFP in the shielding material is necessary in order to achieve a deeper level of comprehension concerning the attenuation performance of the shielding material in relation to the necessary shield thickness. The results of the calculations for the MFP that were performed using the LAC are depicted in Fig. 9. Because it is connected with a shorter MFP, the material with the highest doped material weight percentage demonstrates an ever-increasingly greater capacity for shielding gamma rays. The higher the incident energy, the longer the gamma ray will travel through the shielding material before it is reduced in strength. It is important to note that the shielding effectiveness of the composite is improved when the weight proportion of Bi_2_O_3_ NPs in the composite is increased from 0 to 30%. Numerically, the MFP reduces from 2.048 cm for ERB-0 to 0.337 cm for ERB-30 at 0.06 MeV. For the same composites, the MFP reduces from 7.605 to 5.754 cm at 0.662 MeV. This finding indicated that the manufactured samples with a high level of Bi_2_O_3_ NPs have a promising applicability, particularly for the medical and space sectors, both of which should aim to lower the shield thickness.

**Figure 10:**
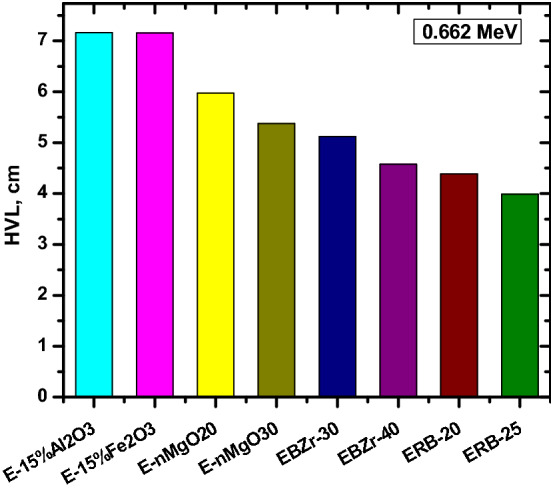
The half value layer for the prepared composites at 0.662 MeV in comparison with other materials.

**Figure 11:**
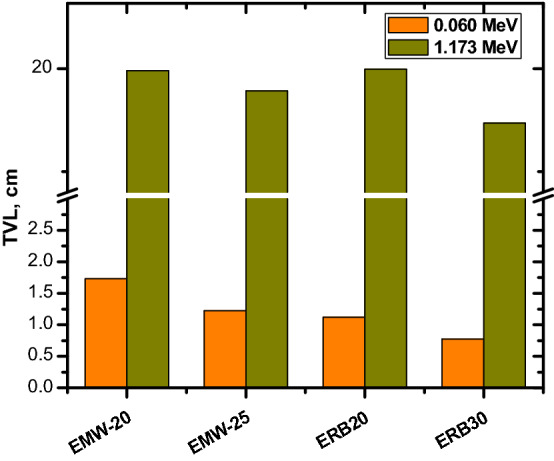
The tenth value layer for the prepared composites in comparison with other materials.

**Figure 12:**
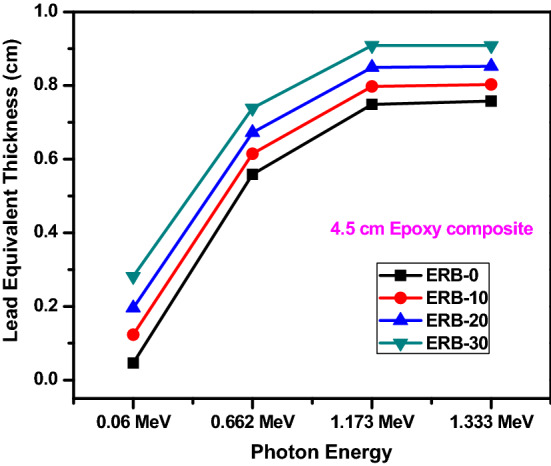
Lead equivalent thickness for the prepared epoxy composites.

We compared the HVL for ERB-20 and ERB-30 with other materials at 0.662 MeV such as E-15%Al_2_O_3_ (85% Epoxy + 15% Al_2_O_3_), E-15%Fe_2_O_3_ (85% Epoxy + 15% Fe_2_O_3_), E-nMgO20 (80% Epoxy + 20% MgO NPs), (70% Epoxy + 30% MgO NPs), EBZ-30 (55% Epoxy + 15% B_2_O_3_ + 30% ZrO_2_), EBZ-40 (45% Epoxy + 15% B_2_O_3_ + 40% ZrO_2_), EMW-20 (50% Epoxy + 30% waste marble + 20% WO_3_) and MW-20 (50% Epoxy + 25% waste marble + 25% WO_3_). The HVL values for ERB-20 and ERB-30 are 4.385 and 3.988 cm and this is lower than all the selected polymers given in Fig. 10. EBZr-40 shows a close HVL with ERB-20.While, from Fig. 11, we can see that the TVL for ERB-20 and ERB-30 at 0.06 and 1.173 MeV are smaller than the TVL for EMW-20 (Epoxy + waste marble + 20% nano WO_3_) and EMW-25 (Epoxy + waste marble + 25% nano WO_3_. Finally, the lead equivalent thickness of the prepared epoxy samples was calculated through the equation found in the literature^43^ as shown in Fig. 12. The results showed that the thickness of 5 cm of the mixture ERB-30 (which is the highest attenuating compound in this work) is equivalent to approximately 1 cm of lead. (Pb) in the attenuation at an energy of 1.333 MeV, while at lower energies such as 0.060 MeV we see that a thickness of 5 cm of ERB-30 mixture is approximately equivalent to 0.3 cm of lead.

## Conclusion

The radiation shielding performance of newly developed epoxy resin with various contents of red clay and Bi_2_O_3_ NPs was reported. We discussed the impact of thickness of the prepared composites as well as the impact of changing the amount of red clay and Bi_2_O_3_ NPs into the radiation shielding parameters for the current composites. We found that the ERB-30 sample had the lowest TF at 0.06 MeV (22.7% for a thickness of 0.5 cm, and 1.2% for a thickness of 1.5 cm.). The TF data demonstrated that the epoxy resin materials have a high attenuation capacity at 0.06 MeV. Regarding the LAC parameter, we found that the incorporating of Bi2O3 NPs enhances this parameter, and the composite which contains 30% of Bi_2_O_3_ NPs has the highest LAC. At 0.662 MeV, increase the Bi_2_O_3_ NPs content by 30% led to 6 times enhancement in the LAC. The Bi_2_O_3_ NPs also affected the TVL, and we found that when the Bi2O3 NPs ratio increases from 0 to 30%, the TVL changes from 4.717 to 0.776 cm at 0.06 MeV and from 17.51 to 13.249 cm at 0.662 MeV. From the MFP results, we concluded that the manufactured samples with a high level of Bi_2_O_3_ NPs have a promising applicability, particularly for the medical and space sectors, both of which should aim to lower the shield thickness.

## Data Availability

All data generated or analyzed during this study are included in this published article.
